# Glucagon-like Peptide-1 Receptor Agonists and Alcohol Use Outcomes: A Systematic Review of Clinical Evidence

**DOI:** 10.3390/jcm15124781

**Published:** 2026-06-19

**Authors:** Ibrahim K. Altami, Eyad A. Alabdulrahim, Osamah M. Alfayez

**Affiliations:** Department of Pharmacy Practice, College of Pharmacy, Qassim University, Buraydah 52571, Saudi Arabia; ibrahimaltami77@gmail.com (I.K.A.); eyad.ahmedar@gmail.com (E.A.A.)

**Keywords:** GLP-1 receptor agonists, alcohol use disorder, alcohol consumption, semaglutide, dulaglutide, systematic review

## Abstract

**Background and Objectives**: Glucagon-like peptide-1 receptor agonists (GLP-1RAs) are widely used for type 2 diabetes and obesity treatment and may influence reward-related behaviors, including alcohol use. This study aimed to evaluate the effects of GLP-1RAs on alcohol consumption and related outcomes in adults with alcohol use or alcohol use disorder (AUD). **Methods**: A systematic review was conducted following PRISMA 2020 guidelines. PubMed and Web of Science were searched from inception to December 2025. Eligible studies included randomized controlled trials (RCTs), secondary analyses of RCTs, and observational studies reporting quantitative alcohol consumption outcomes. Data extraction and risk of bias assessment (RoB 2 and ROBINS-I) were performed independently by two reviewers. **Results**: Five studies (n = 49,892) were included, comprising three RCT-based analyses and one large cohort study. Semaglutide and dulaglutide were associated with modest reductions in alcohol consumption and craving in several studies, with statistically significant improvements in selected behavioral outcomes. In contrast, exenatide did not demonstrate significant effects in the overall AUD population, with signals limited to subgroups. The cohort study showed small but statistically significant reductions in AUDIT-C scores following GLP-1RA initiation. Objective measures (e.g., PEth, breath alcohol concentration) showed reductions in selected contexts but were reported in a few studies. **Conclusions**: GLP-1RAs may be associated with modest reductions in alcohol consumption, but evidence remains limited and heterogeneous. Larger, well-designed RCTs are needed to define their role in the management of AUD.

## 1. Introduction

AUD is a major global public health problem [1]. Alcohol use is causally linked to more than 200 medical and disability conditions, with cardiovascular diseases, liver disease, and multiple cancers contributing substantially to alcohol-related morbidity [2]. AUD is a chronic relapsing condition characterized by neuroadaptations from repeated alcohol exposure that drives compulsive use and addiction [3]. Because the mechanisms driving AUD are heterogeneous, current pharmacological options are limited, and no robust evidence exists to support medication-based controlled drinking strategies [3]. Presently, only a small number of agents—naltrexone, acamprosate, disulfiram, and nalmefene (approved only in Europe)—are licensed for AUD, and many patients either do not respond or cannot tolerate them [4]. Consequently, there remains a need for additional effective pharmacological treatment options for AUD.

Glucagon-like peptide-1 (GLP-1) and glucose-dependent insulinotropic polypeptide are incretin hormones that help regulate blood glucose and have other systemic effects [5]. GLP-1 enhances glucose-dependent insulin release, suppresses glucagon, and reduces appetite and food intake through central and peripheral actions [6]. Experimental work shows that GLP-1 also influences stress, mood, cognition, and reward processing [6]. Because alcohol and other drugs of abuse activate the same reward circuits involved in food reward, GLP-1 signaling is a plausible target within the neural pathways that underlie AUD [7].

Glucagon-like peptide-1 receptor agonists (GLP-1RAs) are approved for the management of type 2 diabetes and are often used for weight control [2,5,8]. They have also been investigated for their effects on alcohol use in both preclinical models and clinical studies [5]. Experimental work indicates that GLP-1RAs reduce voluntary alcohol intake and dampen alcohol reinforcement, supporting their potential clinical application in AUD [2].

Beyond experimental and early clinical studies, emerging real-world data suggest that GLP-1RAs may also reduce alcohol consumption in routine practice [4]. However, these findings are subject to residual confounding and variation in outcome assessment, underscoring the need to systematically appraise and integrate evidence from both randomized trials and real-world studies. Thus, the aim of this systematic review is to evaluate the effects of GLP-1RAs on alcohol consumption and related outcomes in adults with AUD or varying levels of alcohol use.

## 2. Methods

### 2.1. Protocol and Registration

This systematic review was conducted in accordance with the Preferred Reporting Items for Systematic Reviews and Meta-Analyses (PRISMA) 2020 guidelines. The completed PRISMA 2020 checklist is provided in the Supplementary Materials (Supplementary S1). A written protocol was developed before study initiation, but was not prospectively registered in the International Prospective Register of Systematic Reviews (PROSPERO). QuillBot (QuillBot, Chicago, IL, USA) was used for language editing and grammar correction. All content was reviewed and approved by the authors.

### 2.2. Eligibility Criteria

Studies were eligible for inclusion if they involved adult human participants (aged 18 years or older) and evaluated the use of GLP-1RAs in relation to alcohol consumption. Eligible studies included individuals with AUD or varying levels of alcohol use, as well as populations with obesity or metabolic conditions in which alcohol consumption was explicitly measured. Any GLP-1RA agent, dose, route of administration, or treatment duration was considered eligible.

The inclusion criteria consisted of studies reporting quantitative measures of alcohol consumption. These included continuous behavioral outcomes such as heavy drinking days, drinks per drinking day, total alcohol intake, or validated consumption scales (e.g., Alcohol Use Disorder Identification Test-Consumption “AUDIT-C”). Moreover, objective measures of alcohol intake, including phosphatidylethanol (PEth), laboratory alcohol self-administration, or breath alcohol concentration (BrAC), were considered. Eligible study designs included randomized controlled trials (RCTs), secondary analyses of RCTs, and observational studies.

Studies were excluded if they were conducted using animals, were case reports or case series, study protocols without results, conference abstracts without full data, or systematic reviews or meta-analyses. Studies that reported only alcohol-related diagnoses, hospitalizations, liver outcomes, or mortality without quantitative alcohol consumption data were also excluded.

### 2.3. Search Strategy

A comprehensive literature search was conducted in two electronic databases, PubMed and Web of Science Core Collection. The search was performed on 23 December 2025. The search strategy was designed to identify human studies examining the effects of GLP-1RAs on alcohol consumption. Search terms included a combination of keywords and controlled vocabulary related to GLP-1RAs and alcohol consumption or alcohol use.

The GLP-1RA component of the search included terms such as semaglutide, liraglutide, exenatide, dulaglutide, tirzepatide, and lixisenatide, as well as broader terms including glucagon-like peptide-1 receptor agonist, GLP-1, and GLP-1RA. These terms were combined with alcohol-related keywords, including alcohol, alcohol use disorder, AUD, heavy drinking, alcoholism, craving, ethanol, validated consumption measures such as AUDIT-C, and objective measures of alcohol intake such as PEth and BrAC. The search strategy was adapted as needed for each database. Reference lists of included studies were also screened to identify additional relevant articles. Only English-language studies were considered. The complete search strategies for all databases are provided in Supplementary S2.

### 2.4. Study Selection

All records identified through the database searches were first imported into a citation management software (Mendeley), where duplicate records were removed. The remaining unique citations were then uploaded to Rayyan for screening. Titles and abstracts were screened independently by two reviewers to identify studies that potentially met the eligibility criteria. Studies that clearly did not meet the inclusion criteria were excluded at this stage.

Full-text articles were retrieved for all records considered potentially relevant. Full-text screening was also conducted independently by two reviewers. Any disagreements during either stage of screening were resolved through discussion, and when necessary, by consensus with a third reviewer. The number of studies included and excluded at each stage, along with reasons for exclusion at the full-text stage, are reported in the PRISMA flow diagram.

### 2.5. Data Extraction

Data were extracted independently by two reviewers using a standardized data extraction form. Extracted information included study characteristics (such as author, year, country, and study design), participant characteristics (including sample size, age, sex, baseline alcohol consumption, and AUD or obesity status), and details of the intervention and comparator. Information on alcohol consumption outcomes, assessment methods, and timing of outcome measurement was also collected. Any differences in data extraction between reviewers were resolved by discussion and consensus. This approach was used to ensure the accuracy and consistency of the extracted data across all included studies.

### 2.6. Risk of Bias Assessment

Risk of bias was assessed independently by two reviewers. RCTs and secondary analyses of RCTs were assessed using the Cochrane Risk of Bias 2 (RoB 2) tool. The non-randomized cohort study was assessed using the Risk Of Bias In Non-randomized Studies of Interventions (ROBINS-I) tool. Disagreements in risk of bias judgments were resolved through discussion and consensus.

### 2.7. Data Synthesis

Due to substantial clinical and methodological heterogeneity across the included studies, a meta-analysis was not performed. The studies differed with respect to study design, participant populations, GLP-1RA interventions, outcome measures, and follow-up durations. Alcohol-related outcomes were also assessed using diverse subjective and objective measures. Therefore, the findings were synthesized qualitatively.

## 3. Results

### 3.1. Study Selection and Study Characteristics

**Figure 1:**
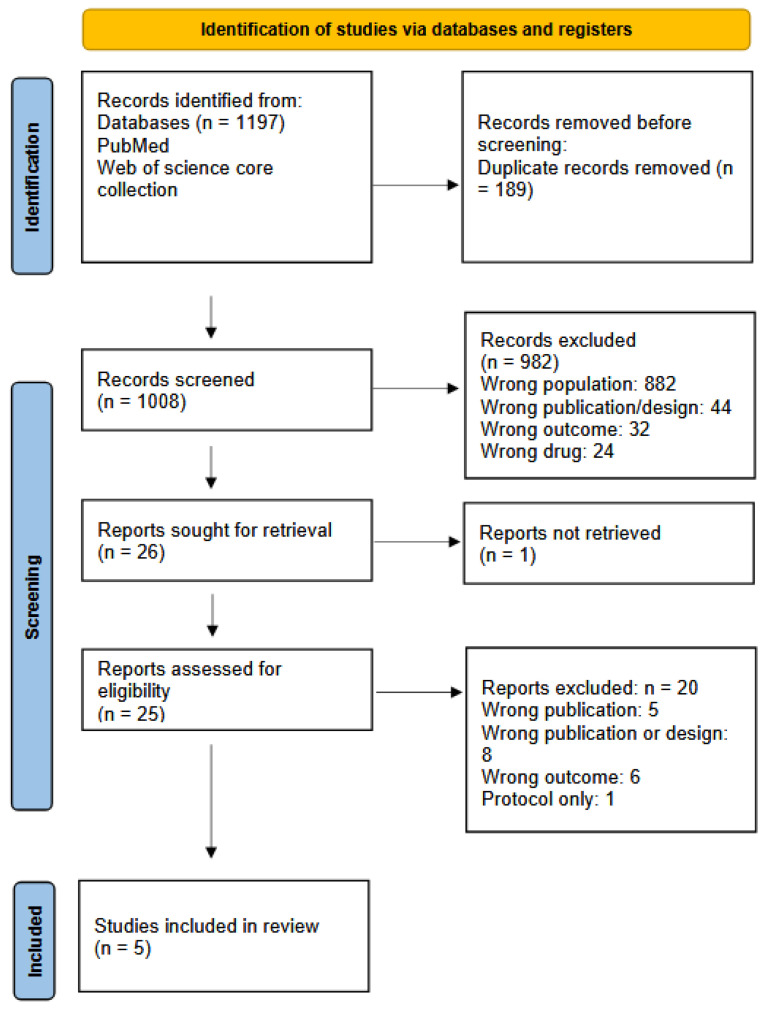
Preferred Reporting Items for Systematic Reviews and Meta-Analyses (PRISMA) flow diagram [9].

The study selection process is summarized in the PRISMA flow diagram (Figure 1). A total of 1197 records were identified through database searches, and 189 duplicates were removed, leaving 1008 unique citations for title and abstract screening. During this stage, 982 records were excluded, most commonly due to ineligible populations (n = 882), inappropriate study design or publication type (n = 44), irrelevant outcomes (n = 32), or evaluation of a non-eligible drug (n = 24). A full-text review was sought for 26 reports, of which one could not be retrieved. The remaining 25 articles underwent a detailed eligibility assessment, resulting in the exclusion of 20 reports. Ultimately, five studies met all inclusion criteria and were included (Figure 1).

Five studies were included: two RCTs, two secondary analyses of randomized trials, and one large real-world cohort study. Sample sizes ranged from 30 to 49,536 participants (Table 1). The studies evaluated exenatide 2 mg weekly subcutaneous, dulaglutide 1.5 mg weekly subcutaneous, and semaglutide up to 1 mg weekly subcutaneous, across treatment-seeking, non-treatment-seeking, smoker, and obesity populations. Comparators included a placebo group, placebo + varenicline, non-exposed clinical controls, and Dipeptidyl Peptidase-4 inhibitor (DPP-4i) group. The duration of the included randomized trials ranged from 9 to 26 weeks. Longitudinal data were supplemented by a 24-week follow-up visit in the Klausen study [7], and an extended observational period of approximately 36 weeks in the Farokhnia study [4]. Alcohol outcomes were assessed using AUDIT-C, heavy drinking days, drinks per week, lab self-administration + drinking metrics, Timeline Follow-Back (TLFB), and PEth (Table 1).

### 3.2. Baseline Patient Characteristics

Baseline characteristics varied substantially across studies. The included populations comprised 49,892 participants, with the majority drawn from the Farokhnia study [4]. Participants in the Farokhnia study were older, with the most common age band between 60 and 69 years [4], whereas the remaining four studies’ populations were younger, with mean ages ranging from approximately 40 to 53 years. Sex distribution differed by study. Women represented approximately 7% of the Farokhnia study’s population [4], whereas they accounted for 30% to 71% of participants in the remaining four studies. Weight was not reported in the studies by Farokhnia [4] and Probst [8]. The remaining studies reported mean body weights ranging from 82.5 to 102.8 kg. Mean Body Mass Index (BMI) was not reported in the Farokhnia study [4] and the Probst study [8]; on the other hand, the remaining three studies reported a mean BMI ranging from 26.7 to 33.7. However, obesity prevalence ranged from 90.8% in the Probst study to 100% in the Jensen study [8,10]. Type 2 diabetes mellitus was not reported in the Farokhnia study [4], whereas it was excluded in the remaining four studies. Smoking prevalence varied, all participants were smokers in the Probst study, and 27% were active smokers in the Hendershot study (Table 2) [2,8].

### 3.3. Baseline Alcohol Profile Summary

Baseline alcohol severity differed across studies and reflected heterogeneous drinking patterns. Alcohol consumption was assessed using a range of validated measures, including TLFB, weekly consumption estimates, laboratory alcohol self-administration, AUDIT/AUDIT-C, craving scales, drinks per drinking/calendar day, heavy drinking days, days without alcohol consumption, total alcohol consumption, and the PEth biomarker (Table 3). In the Klausen study, participants with treatment-seeking AUD had high dependence severity, with AUDIT scores in the mid-20s and frequent heavy drinking days over 30 days [7]. Probst enrolled smokers with comparatively low to moderate alcohol use, with a median of 3 drinks per week and only about 12% classified as heavy drinkers [8]. Farokhnia stratified participants by AUDIT-C into low-risk, at-risk, and hazardous/binge-drinking categories, with most falling into the low-risk group [4]. In Hendershot’s study, non-treatment-seeking adults with AUD showed moderate baseline severity (AUDIT around 13) with regular heavy drinking and moderate craving scores [2]. Jensen focused on an obese AUD subgroup with high dependence severity, AUDIT scores in the mid-20s, frequent heavy drinking days, and elevated PEth levels at baseline [10] (Table 3).

### 3.4. Behavioral Alcohol Outcomes

Behavioral alcohol outcomes were reported in four of the five included studies using self-reported measures and standardized tools, including drinks per week, drinks per drinking/calendar day, heavy drinking days, total alcohol consumption, abstinent days, craving scales, and AUDIT/AUDIT-C scores. In the randomized trial by Klausen, exenatide did not significantly reduce heavy drinking days or other behavioral alcohol outcomes compared with placebo in the overall AUD population [7]. In the secondary analysis by Probst, dulaglutide significantly reduced weekly alcohol consumption compared with the placebo; by 12 weeks, alcohol intake was approximately 29% lower in the dulaglutide group (*p* = 0.04) [8]. In the cohort study by Farokhnia, GLP-1RA initiation was associated with significantly greater reductions in AUDIT-C scores compared with both unexposed individuals (*p* = 0.0025) and DPP-4i users (*p* = 0.0002) [4]. In the randomized trial by Hendershot, once-weekly semaglutide significantly reduced drinks per drinking day compared with placebo (*p* = 0.04), and total alcohol consumption and weekly cravings were both significantly reduced (each *p* = 0.01). Furthermore, significant reductions in heavy drinking days were noted over time (*p* = 0.04), whereas drinks per calendar day did not differ significantly between groups [2].

### 3.5. Objective Alcohol Outcomes

Objective alcohol outcomes were reported in three studies using validated measures, including the PEth biomarker and laboratory measures of mean and peak BrAC. In the randomized trial by Klausen, PEth levels did not differ significantly between the exenatide and placebo group over the 26-week treatment period (*p* = 0.64) [7]. In contrast, the secondary analysis by Jensen, restricted to participants with BMI ≥ 30 kg/m^2^, found a statistically significant reduction in PEth at week 26 in the exenatide group compared with placebo (*p* = 0.03), with no significant between-group differences at earlier time points [10]. In the randomized trial by Hendershot, semaglutide produced statistically significant reductions in both mean and peak BrAC during the post-treatment self-administration session compared with the placebo (*p* = 0.02 and *p* = 0.03, respectively) [2]. Overall, these objective measures provide converging evidence of reduced alcohol exposure with GLP--1RA treatment in specific contexts, but effects have been demonstrated in a limited number of studies and at select time points only.

### 3.6. Risk of Bias Within Studies

Risk of bias was assessed using the Cochrane Risk of Bias 2 (RoB 2) tool for randomized controlled trials and the ROBINS--I tool for the non-randomized cohort study (Table 4A,B). Among the four randomized studies, one trial (Hendershot) was judged to be at low risk of bias, whereas three (Klausen, Probst, Jensen) had some concerns, mainly due to missing outcome data and issues related to secondary or subgroup analyses and selection of reported outcomes. The observational Farokhnia cohort was judged to have a moderate overall risk of bias, reflecting potential residual confounding and outcome measurement limitations inherent to self-reported AUDIT-C, despite extensive propensity score matching and sensitivity analyses. The body of evidence was considered to have acceptable internal validity, with greater confidence placed in the findings from randomized trials.

## 4. Discussion

In this systematic review, we identified five clinical studies evaluating GLP-1RAs in relation to alcohol consumption and related outcomes in adults with alcohol use or diagnosed AUD. Several RCTs and secondary analyses showed that semaglutide or dulaglutide were associated with modest reductions in alcohol craving and consumption compared with the placebo, with statistically significant improvements reported in selected outcomes [2,8,10]. In contrast, one RCT of exenatide in treatment-seeking patients with AUD did not show significant benefits on drinking or craving in the overall sample, with only exploratory signals in participants with obesity [7]. A large real-world cohort study reported small but statistically significant reductions in AUDIT-C scores following GLP-1RA initiation compared with unexposed and active comparator groups [4].

The included studies varied in populations, agents, and endpoints, which may explain the observed heterogeneity. Across the examined studies, semaglutide and dulaglutide were generally associated with reductions in alcohol consumption and related outcomes, whereas exenatide showed limited effects in treatment-seeking AUD populations [2,7,8]. These findings suggest that treatment effects may vary by population characteristics and outcome assessment methods. More consistent signals were observed in studies using detailed or repeated measures of alcohol consumption and in non-treatment-seeking populations; however, these findings are based on a small number of studies and should be interpreted with caution.

These findings are consistent with recent systematic reviews and meta-analyses evaluating GLP-1RAs and alcohol-related outcomes. Several analyses report modest but directionally similar reductions in alcohol consumption, heavy drinking days, and craving, although the results are heterogeneous across study designs and populations [11,12,13,14]. Sinha and colleagues found small, non-significant average effects on consumption and craving across three RCTs [12], contrasted with more pronounced associations for alcohol-related events in large observational cohorts. Eshraghi et al. reported overall reductions in AUDIT scores and alcohol intake but with substantial heterogeneity across studies [14].

Our findings align with this pattern. Semaglutide and dulaglutide were associated with reductions in alcohol consumption in selected settings, while exenatide showed limited effects in the overall AUD population. The large cohort study also demonstrated small but statistically significant reductions in AUDIT-C scores following GLP-1RA initiation [4]. Beyond consumption outcomes, pharmacoepidemiologic studies have reported lower rates of alcohol-related events and incident AUD diagnoses among GLP-1RA users [15,16,17]. However, these outcomes were outside the scope of this review and should be interpreted cautiously, given residual confounding and variability in endpoint definitions. Importantly, the included studies evaluated different GLP-1RA, including exenatide, dulaglutide, and semaglutide, which differ in pharmacokinetic and pharmacodynamic properties. Although semaglutide demonstrated the most consistent reductions in alcohol-related outcomes, the current evidence is insufficient to determine whether these findings reflect agent-specific effects or differences in study populations and outcome measures.

The observed patterns in alcohol-related outcomes may be explained by the central effects of GLP-1RAs on reward processing and motivation. GLP-1 receptors are expressed in key brain regions involved in addiction, including the ventral tegmental area and nucleus accumbens, where they modulate dopaminergic signaling and reduce reward sensitivity [13,18]. These mechanisms may help explain the consistent reductions in drinking intensity and craving observed in some trials, as well as the more pronounced effects in individuals with higher baseline alcohol use or coexisting metabolic disease [19]. The greater signal seen with semaglutide and dulaglutide compared with exenatide may reflect differences in pharmacokinetics and central receptor engagement, although direct comparative evidence is limited [11]. These mechanisms provide a plausible biological basis for the clinical findings. However, the extent to which these pathways translate into sustained reductions in alcohol consumption in humans remains uncertain and requires further investigation.

The GLP-1RA class comprises pharmacologically distinct agents rather than a homogeneous group, which may contribute to differences in their effects on alcohol-related outcomes. Exenatide is a short-acting GLP-1RA with a relatively short half-life, whereas semaglutide is a long-acting analog with strong albumin binding and prolonged systemic exposure. These pharmacokinetic differences result in more sustained receptor activation with semaglutide compared with exenatide. In addition, GLP-1RAs differ in receptor affinity, potency, and central nervous system exposure, factors that may influence their effects on reward-related pathways implicated in alcohol use disorder. Although the available evidence is insufficient to determine whether these pharmacological differences translate into clinically meaningful differences in AUD outcomes, they may partially explain why findings appeared more favorable with semaglutide than with exenatide in the studies included in this review. Further mechanistic and comparative clinical studies are needed to clarify whether specific GLP-1RAs offer advantages for reducing alcohol consumption and craving.

This review has several limitations. First, the number of included studies was small, which limits the strength of inference. The available RCTs were limited in sample size and duration, and some were secondary analyses. Publication bias cannot be excluded, and unpublished studies with negative or neutral findings may have influenced the overall evidence base. Second, there was substantial heterogeneity across studies in populations, GLP-1RA agents, doses, and alcohol-related outcomes, which limited direct comparisons and prevented quantitative synthesis. Third, alcohol outcomes were measured using different tools, and objective measures were reported in only a few studies. Fourth, the observational cohort study may be subject to residual confounding despite adjustment methods. Fifth, prior to study initiation, a written protocol was developed and adhered to throughout the review process. The protocol was not, however, prospectively registered in PROSPERO or another public registry. Methodological transparency may decrease and the external validation of compliance with the original protocol is limited, which can increase the risk of selective reporting. Sixth, a formal certainty-of-evidence assessment using the GRADE framework was not performed. Consequently, the overall strength and certainty of the available evidence could not be formally evaluated and should be interpreted with caution. Finally, the literature search was limited to PubMed and Web of Science and did not include additional databases such as Embase, Scopus, or Cochrane CENTRAL, nor gray literature sources. Therefore, some potentially relevant studies may not have been identified. Also, this review was limited to published data and English language studies. Overall, the available evidence should be considered preliminary and interpreted with caution.

GLP-1RAs may represent a promising adjunct for reducing alcohol consumption, particularly in patients with coexisting metabolic conditions such as obesity or type 2 diabetes. However, the current evidence is not sufficient to support their routine use as primary treatment for AUD. Clinicians should consider potential effects on alcohol use when initiating GLP-1RAs for metabolic indications and monitor patients accordingly. Future studies should focus on adequately powered RCTs in patients with AUD, with longer follow-up and prespecified alcohol-related endpoints. Standardized outcome measures and inclusion of objective biomarkers are needed to improve comparability across studies. Comparative trials across GLP-1RA agents and inclusion of more diverse populations will also be important.

## 5. Conclusions

GLP-1RAs were associated with modest reductions in alcohol consumption and craving in several studies, but findings were inconsistent across outcomes and populations. The current evidence is limited and heterogeneous, with more consistent signals observed in observational data than in RCTs. The available evidence remains preliminary, and larger, well-designed RCTs are needed to clarify the role of GLP-1RAs in the management of AUD and to determine whether they have a place in clinical practice.

## Figures and Tables

**Table 1:** Study characteristics.

Variable/Study	Klausen 2022 [7]	Probst 2023 [8]	Farokhnia 2025 [4]	Hendershot 2025 [2]	Jensen 2025 [10]
Country/Setting	Denmark; addiction clinics	Switzerland; hospital smoking clinic	USA; VA EHR	USA; academic outpatient + lab	Denmark; addiction clinics
Design	Randomized, double-blinded, placebo-controlled clinical trial	Predefined secondary analysis of a randomized, double blinded, placebo-controlled trial	Cohort (propensity-matched)	Phase 2 randomized, double-blinded, placebo-controlled clinical trial	Secondary analysis of a randomized, double blinded, placebo-controlled trial
Population	Treatment-seeking AUD adults	Smokers; alcohol consumers subgroup	Veterans receiving GLP-1RAs; alcohol use assessed via AUDIT-C	Non-treatment-seeking AUD adults	AUD + obesity subgroup
Sample size (analyzed)	n = 127	n = 151	GLP-1RA vs. unexposed n = 26,528	n = 48	n = 30
			GLP-1RA vs. DPP-4i = 23,008		
GLP-1RA agent and dose	Exenatide 2 mg weekly	Dulaglutide 1.5 mg weekly	Routine clinical GLP-1RAs	Semaglutide up to 1 mg weekly	Exenatide 2 mg weekly
Comparator	Placebo	Placebo + varenicline	Unexposed controls (and DPP-4i)	Placebo	Placebo
Treatment duration	26 weeks	12 weeks	Variable prescribing exposure	9 weeks	26 weeks
Follow-up (Outcome window)	Long-term 24 week follow-up visit	12 weeks	~36 weeks	9–10 weeks	26 weeks
Alcohol outcomes	Heavy drinking days, TLFB	Drinks/week	AUDIT-C change	Lab self-administration + drinking metrics	PEth + TLFB

Abbreviations: AUD = alcohol use disorder; AUDIT-C = Alcohol Use Disorders Identification Test–Consumption; EHR = electronic health record; GLP-1RA = glucagon-like peptide-1 receptor agonist; PEth = phosphatidylethanol; TLFB = Timeline Follow-Back; VA = United States Department of Veterans Affairs; USA = United States of America; DPP-4i = Dipeptidyl Peptidase-4 inhibitor. Comments: Follow-up refers to the time window during which alcohol outcomes were assessed and may differ from treatment duration. Farokhnia 2025 [4] represents real-world pharmacoepidemiologic data, whereas the remaining studies are clinical trials. Probst 2023 [8] is the predefined secondary analysis of randomized controlled trials. Jensen 2025 [10] is the secondary analysis of randomized controlled trials. Alcohol outcome measures varied across studies (e.g., AUDIT-C, TLFB, PEth, and laboratory alcohol self-administration).

**Table 2:** Baseline demographics and clinical profile of included studies.

Variable/Study	Klausen 2022 [7]	Probst 2023 [8]	Farokhnia 2025 [4]	Hendershot 2025 [2]	Jensen 2025 [10]
Sample analyzed (n)	127	151	GLP-1RA vs. unexposed (14,130 vs. 12,398 matched participants)	48	30
			GLP-1RA vs. DPP-4i (11,863 vs. 11,145 matched participants)		
Age ± SD (years)	52.3 ± 10.4	42 (median, IQR 33–53)	60–69 years most common age band	39.9 ± 10.6	53
Female sex (%)	40%	61%	6.6% *	71%	30%
Weight ± SD (kg)	82.5 ± 17.2	NR	NR	94.2 ± 18.1	102.8 ± 13.6
BMI mean ± SD (kg/m^2^)	26.7 ± 4.9	NR	NR	32.1 ± 5.6	33.7 ± 3.3
Obesity (BMI ≥ 30) (n, %)	NR	119 (90.8)	NR	NR	30 (100)
Smoking n (%)	NR	151 (100)	NR	13 (27)	NR

Abbreviations: NR = not reported; BMI = Body Mass Index; T2DM = type 2 diabetes mellitus; SD standard deviation; GLP-1RA: Glucagon-Like Peptide-1 Receptor Agonist; DPP-4i: Dipeptidyl Peptidase-4 inhibitor; IQR: Interquartile Range. Comments: The values reflect baseline characteristics of participants included in alcohol outcome analyses. * Calculated from the matched analytic cohorts with follow-up AUDIT-C in the original publication (GLP-1RA vs. unexposed and GLP-1RA vs. DPP-4i comparisons). The proportion of female participants was calculated using the total number of female participants divided by the total number of matched participants across both cohorts.

**Table 3:** Baseline alcohol profile of included studies.

Variable (Baseline)	Klausen 2022 [7]	Probst 2023 [8]	Farokhnia 2025 [4]	Hendershot 2025 [2]	Jensen 2025 [10]
Alcohol Measures	TLFB + heavy drinking days	Weekly drinks (self-report)	AUDIT-C	Primary outcome: Laboratory alcohol self- administration. Secondary outcome: TLFB + Alcohol craving and Alcohol consumption.	TLFB + PEth
Alcohol severity metric (AUDIT or AUDIT-C scores)	Exenatide group: 25.6 ± 5.7 Placebo group: 25.9 ± 5.2	NR	NR	13.4 ± 6.0	Exenatide group: 26.7 ± 4.3 Placebo group: 22.3 ± 3.7
Drinks (calendar day)	NR	NR	NR	2.9 ± 1.7	NR
Drinks (drinking day)	NR	NR	NR	4.2 ± 2.2	NR
Heavy drinking days (30 days)	Exenatide group: 16.7 ± 8.2 Placebo group: 17.3 ± 8.5	NR	NR	9.1 ± 6.8	Exenatide group: 19.14 ± 7.68 Placebo group: 15.27 ± 8.25
Total alcohol consumption	Exenatide group: 2370 ± 1580 Placebo group: 2430 ± 1860	NR	NR	NR	Exenatide group: 3097.3 ± 1931.5 Placebo group: 1935.8 ± 1509
Days without alcohol consumption (30 days)	Exenatide group: 9.11 ± 7.3 Placebo group: 9.92 ± 7.9	NR	NR	NR	NR
Alcohol craving	NR	NR	NR	PACS: 12.0 ± 5.6	NR
Biomarker (PEth)	NR	NR	NR	NR	Exenatide group: 1.1 ± 0.5 μmol/L Placebo group: 1.2 ± 1.7 μmol/L

Abbreviations: AUDIT = Alcohol Use Disorders Identification Test; AUDIT-C = Alcohol Use Disorders Identification Test–Consumption; TLFB = Timeline Follow-Back; PACS = Penn Alcohol Craving Scale; PEth = phosphatidylethanol; NR = not reported; IQR = Interquartile Range. Comments: For Jensen et al., heavy drinking days were estimated by multiplying the reported percentage of heavy drinking days by 30 days.

**Table 4:** Risk of bias assessment of included studies.

(**A**) **Risk of Bias Assessment of Included Randomized Controlled Trials (RoB 2)**								
**Study**	**Randomization Process**		**Deviations from Intended Interventions**	**Missing Outcome Data**	**Measurement of the Outcome**		**Selection of the Reported Result**	**Overall Risk of Bias**
Hendershot et al., 2025 [2]	Low risk		Low risk	Low risk	Low risk		Low risk	Low risk
Klausen et al., 2022 [7]	Low risk		Low risk	Some concerns	Low risk		Some concerns	Some concerns
Probst et al., 2023 [8]	Low risk		Low risk	Some concerns	Low risk		Some concerns	Some concerns
Jensen et al., 2025 [10]	Low risk		Low risk	Some concerns	Low risk		Some concerns	Some concerns
(**B**) **Risk of Bias Assessment of the Observational Study Using ROBINS-I**								
**Study**	**Bias Due to Confounding**	**Bias in the Selection of Participants**	**Bias in the Classification of Interventions**	**Bias Due to Deviations from Intended Interventions**	**Bias Due to Missing Data**	**Bias in the Measurement of Outcomes**	**Bias in the Selection of the Reported Result**	**Overall Risk of Bias**
Farokhnia et al., 2025 [4]	Moderate	Low	Low	Low	Low	Moderate	Low	Moderate

Abbreviations: RoB 2: Cochrane Risk of Bias 2 tool, ROBINS-I: Risk Of Bias In Non-randomized Studies of Interventions.

## Data Availability

All data generated or analyzed during this paper are included in this published article.

## Supplementary Materials

The following supporting information can be downloaded at https://www.mdpi.com/article/10.3390/jcm15124781/s1, Supplementary S1: PRISMA 2020 checklist [9]. Supplementary S2: Complete Search Strategies.
