# Supplementary material for: Glucagon-like Peptide-1 Receptor Agonists and Alcohol Use Outcomes: A Systematic Review of Clinical Evidence
Source: J Clin Med. 2026 Jun 19;15(12):4781. doi: 10.3390/jcm15124781 (PMC13300848; doi:10.3390/jcm15124781)
Supplement: Supplementary file 1 [file jcm-15-04781-s001.zip › jcm-4351833-supplementary.pdf]

Supplementary S2. Complete Search Strategies

Search Date: 23 December 2025

The search strategy was designed to identify human studies evaluating glucagon-like peptide-1 receptor agonists (GLP-1RAs) and alcohol-related outcomes. Search terms included individual GLP-1RA agents, class-related terminology, alcohol consumption measures, alcohol use disorder terminology, and objective alcohol biomarkers. Search strategies were adapted to the syntax and indexing structure of each database.

1. PubMed

Search Strategy

((((((((((((((((((("semaglutide"[All Fields]) OR (semaglutide)) OR ("liraglutide"[All Fields])) OR (liraglutide)) OR ("exenatide"[All Fields])) OR (exenatide)) OR ("dulaglutide"[All Fields])) OR (dulaglutide)) OR (tirzepatide)) OR ("tirzepatide"[All Fields])) OR ("lixisenatide"[All Fields])) OR (lixisenatide)) OR ("glucagon like peptide 1 receptor agonist"[All Fields])) OR (glucagon like peptide 1 receptor agonist)) OR ("glp 1"[All Fields])) OR ("glucagon like peptide 1"[All Fields])) OR ("glp 1ra"[All Fields]))

AND

((((((((((((((((((((((((("alcohol"[All Fields]) OR (alcohol)) OR ("alcohol use disorder"[All Fields])) OR (alcohol use disorder)) OR ("aud"[All Fields])) OR ("alcoholism"[All Fields])) OR (alcoholism)) OR ("heavy drinking"[All Fields])) OR (heavy drinking)) OR ("alcoholic"[All Fields])) OR (alcoholic)) OR ("craving"[All Fields])) OR ("ethanol"[All Fields])) OR ("audit c"[All Fields])) OR ("alcohol use disorder identification test"[All Fields])) OR ("alcohol use disorder identification test audit"[All Fields])) OR (alcohol use disorder identification test)) OR ("phosphatidylethanol"[All Fields])) OR (phosphatidylethanol)) OR ("peak breath alcohol concentration"[All Fields])) OR ("peak breath alcohol concentration")) OR (BrAC)) OR (PEth))

NOT

((((((("case report"[All Fields]) OR ("review"[All Fields])) OR ("systematic review"[All Fields])) OR ("meta-analysis"[All Fields])) OR ("meta-analyses"[All Fields])) OR ("animal"[All Fields])) OR ("case series"[All Fields]))

Filters Applied

• Humans
• English language

2. Web of Science Core Collection

Search Strategy

TS=(
("semaglutide" OR "liraglutide" OR "exenatide" OR "dulaglutide" OR
"tirzepatide" OR "lixisenatide" OR
"glucagon like peptide 1 receptor agonist" OR
"GLP-1" OR "GLP-1RA")
AND
("alcohol" OR "alcohol use disorder" OR "AUD" OR
"alcoholism" OR "heavy drinking" OR "alcoholic" OR
"craving" OR "ethanol" OR "AUDIT-C" OR
"Alcohol Use Disorders Identification Test" OR
"phosphatidylethanol" OR "PEth" OR
"breath alcohol concentration" OR "BrAC")
)

NOT

TS=("case report" OR "case series" OR "review" OR
"systematic review" OR "meta-analysis" OR
"animal")

Refinements Applied

- Document type: Article
- Language: English

3.Additional Search Methods

The reference lists of all included studies were manually screened to identify any additional eligible studies not captured by the electronic database searches.
